# Treatment with Growth Hormone (GH) Increased the Metabolic Activity of the Brain in an Elder Patient, Not GH-Deficient, Who Suffered Mild Cognitive Alterations and Had an ApoE 4/3 Genotype

**DOI:** 10.3390/ijms19082294

**Published:** 2018-08-05

**Authors:** Jesús Devesa, Iria Núñez, Carlos Agra, Alejandro Bejarano, Pablo Devesa

**Affiliations:** 1 Scientific Direction, Medical Center Foltra, Travesía de Montouto 24, 15886 Teo, Spain; 2 Nuclear Medicine, Hospital HM Modelo, Virrey Osorio 30, 15011 Coruña, Spain; inunez@hmhospitales.com (I.N.); abejarano@hmhospitales.com (A.B.); 3 Neuropsychology, Medical Center Foltra, Travesía de Montouto 24, 15886 Teo, Spain; carlosagra80@gmail.com; 4 Research and Development, Medical Center Foltra, Travesía de Montouto 24, 15886 Teo, Spain; pdevesap@gmail.com

**Keywords:** Growth hormone, cognition, recent memory, PET-SCAN, hippocampus, amygdala, parahippocampus, ApoE genotype

## Abstract

(1) Background: We analyzed, using PET-SCAN and cognitive tests, how growth hormone (GH) could act in the brain of an older woman, not deficient in GH, who showed mild cognitive alterations (MCI) and had a genotype of ApoE 4/3 and familial dyslipidemia. (2) Methods: After performing a first psychometric study (TAVEC verbal learning test), the metabolic activity of brain structures related to knowledge, memory, and behavior was analyzed using 18-F fluorodeoxyglucose PET-SCAN. The patient was then treated with GH (0.4 mg/day, subcutaneous) for three weeks and on the last day under this treatment, a new PET-SCAN was performed. One month after beginning treatment with GH, a new TAVEC test was performed. (3) Results: GH administration normalized the cognitive deficits observed in the first psychometric test and significantly (*p* < 0.025) increased the metabolic activity in practically all brain cortical areas, specifically in the left hippocampus and left amygdala, although not in the left parahippocampus. (4) Conclusions: This study demonstrates for the first time the positive effects of GH on cerebral metabolism in a patient without GH deficiency, recovering the function of affected areas related to knowledge, memory, and behavior in an elderly patient with MCI.

## 1. Introduction

In 1993, it was discovered that the growth hormone (GH) receptor gene was expressed in many different areas of the central nervous system (CNS) of rats and rabbits [1], a finding confirmed three years later when it was described that not only the GH receptor (GHR), but also the hormone itself was present in the CNS [2]. These findings led to the assumption that GH should perform important functions at the central level, beyond the classical concept that GH is only a pituitary hormone whose actions take place at the metabolic level and on the longitudinal growth of the organism before puberty is finished. In fact, the current concept is that GH is a pleiotropic hormone, which is expressed in virtually all tissues and organs in which, in addition to endocrine effects, it exerts a number of auto- and paracrine actions [3,4].

GH plays a key role in the organization of the brain during the development of the CNS [5,6,7], and also in its normal functioning after birth, both in animals and humans. It has been found that the hormone is produced in the postnatal hippocampus in rats [8,9], probably to induce proliferation and survival of neuronal precursors at this level, as occurs after the administration of GH in normal adult rats [10]; this also occurs, in rats, when the hormone is given after a brain injury produced by the administration of kainic acid administration, perhaps cooperating with the endogenously expressed GH, as its receptor, in progenitor cells of the hippocampus [11]. In addition, memory tasks induce the synthesis of GH in the hippocampus of mice, which leads to the appearance of newly formed neurons [12]. All this suggests that GH plays an important role in the hippocampus, a structure directly related to the acquisition of recent memory. It is well known that human adults with GH deficiency (GHD) show clear psychological improvements when treated with the hormone [2], particularly in aspects related to memory and cognition [13]. On the contrary, adults with GHD who do not receive replacement therapy usually show significant psychological alterations, such as lack of energy, impaired memory, and cognitive alterations [14,15]. These effects of GH on cognitive functions in humans have been widely reviewed recently [4,16]. However, it is not known if they occur as a direct action of the hormone at the central level, or if they depend on a higher production of hepatic IGF-I induced by GH (or directly induced by the hormone in the CNS), or if those actions are a consequence of the effects of both hormones [4,17,18]. In fact, it has been shown that GH induces the local expression of IGF-I in the human fetal cortex [19], although this has not yet been demonstrated in the human adult brain. The pituitary secretion of GH reaches very high levels at puberty, but once it ends, a gradual decrease in the secretion of the hormone begins, starting from 18 to 30 years, until the plasma levels of GH are virtually undetectable as we get older [4,17]. Therefore, it seems logical that aging is associated with a cognitive deterioration, produced, among other factors, by a deficit of the GH/IGF-I axis [17,18].

In this study, we will describe the effects of short-term GH treatment (0.4 mg/day, 3 weeks) on the cognitive deficits of an elderly woman. Given that she had a family history of Alzheimer’s disease, in addition to a cognitive test (TAVEC verbal learning test), we visualized through PET-SCAN the metabolic activity of her brain, before and after the administration of GH. The data obtained in this study indicate that initially there was a clear decrease in the metabolic activity of the left hippocampus, left amygdala, and left parahippocampus, but these hypometabolisms improved significantly, with the exception of the left parahippocampus, after treatment with GH. In addition, a clear increase in metabolic activity was observed in virtually all cerebral cortical areas. These brain metabolic changes were accompanied by positive changes in a new cognitive TAVEC test, and also reported by the patient herself.

## 2. Results

### 2.1. Cognitive Test: TAVEC Test

Most of the values recorded as deficient in the first test changed to a mean value for a normal population during the second test performed. This indicates a positive learning curve, as well as increased attention. Likewise, the discrimination index also indicated learning on the part of the patient, because in the second test she was already able to store the information in a discriminatory way. Also, during the second test performed, there was no loss of information (fading) over time.

These data indicate that the treatment with GH induced an improvement in learning, attention, and memory, although due to the short time elapsed between the two tests, no statistical analysis of the results obtained was performed.

The scores of this test are shown in Table 1.

### 2.2. PET-SCAN Studies

Treatment with GH led to an overall qualitative and quantitative increase in the metabolic activity observed in most cortical brain regions in both hemispheres.

When evaluated using the software described in Methods, the first PET-SCAN showed significant hypometabolism (Table 2) in the following regions of interest (ROI): the left hippocampus (Z-score = −2.79) and the left amygdala (Z-score = −2.23), the left parahippocampus (Z-score = −2.12), the left cuneus (Z-score = −2.06), and the subgenual area of the anterior cingulate cortex (Z-score = −2.17) of the right hemisphere. These deficits, except for the left cuneus, were 1.5 standard deviations (SD) lower than the mean value registered in the database of the normal population, and clearly asymmetric with respect to the metabolic activity detected in the other hemisphere. These findings were corroborated by the statistical parametric mapping (SPM) analysis (see Methods), which confirmed that these hypometabolisms were statistically significant (*p* < 0.025).

These hypometabolisms were normalized after treatment with GH, as indicated in Table 2, with the sole exception of the parahippocampus, which in the analysis based on ROI still showed a decrease in the acquisition of fluorodeoxyglucose (FDG) (Z-score = −2.04).

**Figure 1:**
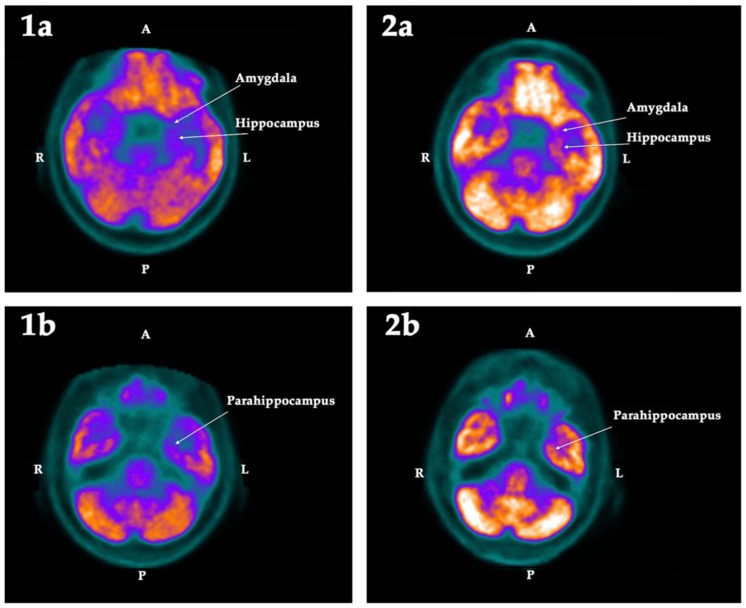
Cross section of the brain showing the metabolic activity in the left amygdala and left hippocampus (**1a**) and the left parahippocampus (**1b**), in the first (1) and the second (2) PET-SCAN studies. Note that the low metabolic activity in the structures observed in the first PET-SCAN was normalized in the left amygdala and the left hippocampus (*p <* 0.025) after treatment with GH (**2a**), but it was not statistically significant in the left parahippocampus (**2b**). A: Anterior. R: Right. L: Left. P: Posterior.

**Figure 2:**
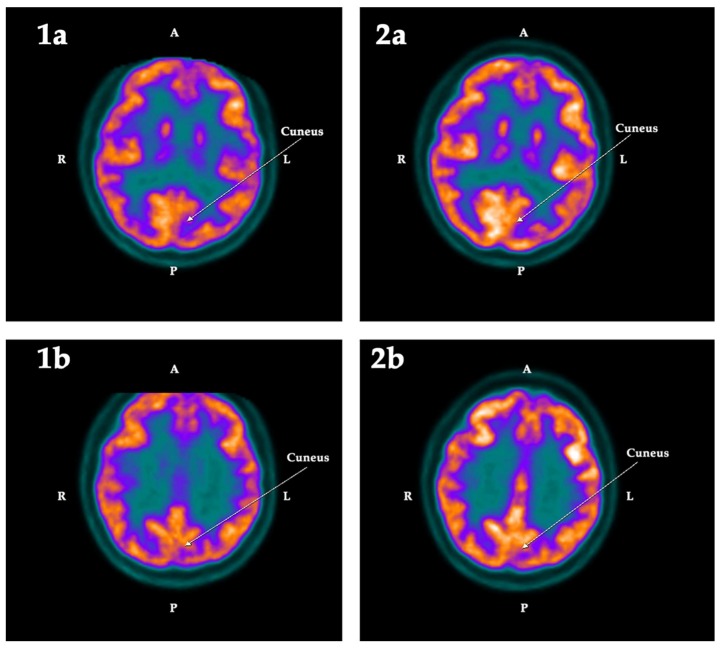
Cross section of the brain showing, in two consecutive sections, the metabolic activity in the left cuneus in the first (1) and the second (2) PET-SCAN studies. Note that the low metabolic activity observed in the left cuneus in the first PET-SCAN (**1a**,**1b**) was normalized (*p* < 0.025) after treatment with GH (**2a**,**2b**). A: Anterior. R: Right. L: Left. P: Posterior.

**Figure 3:**
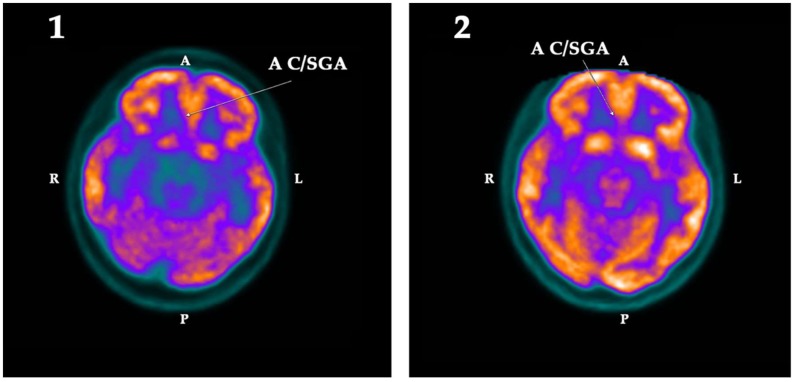
Cross section of the brain showing the metabolic activity in the subgenual area of the anterior cingulate cortex (A C/SGA) in the first (**1**) and the second (**2**) PET-SCAN studies. Note that the low metabolic activity observed in A C/SGA in the first PET-SCAN was normalized (*p* < 0.025) after treatment with GH. A: Anterior. R: Right. L: Left. P: Posterior.

The qualitative changes in the metabolism observed in these regions are shown in the following Figure 1, Figure 2 and Figure 3:

### 2.3. Blood Analysis

The blood test prior to the treatment showed that the patient had dyslipidemia (total cholesterol plasma values: 275 mg/dL (normal: 110–200 mg/dL), plasma triglycerides: 195 mg/dL (normal: 50–150 mg/dL). The erythrocytes (4.95 × 10^6^/µL) and hemoglobin (Hb) (14.2 g/dL) and leukocytes were in normal values, as were the values of plasma glucose (94.8 mg/dL), proteins (total proteins: 7.1 g/dL; albumin: 4.3 g/dL), liver transaminases, urea, creatinine, and tumor markers (CA-125, CA15-3, CA19-9, alpha-fetoprotein, and CEA). Plasma thyroid stimulating hormone (TSH) was normal (2.18 μUI/mL), as was free thyroxine (fT4, 1.1 ng/dL); plasma cortisol at 08.00 h was also normal (24 μg/dL; normal values: 8–25 µg/dL). Plasma IGF-I values (125 ng/mL) and plasma insulin-like growth factor-binding protein 3 (IGFBP3: 2.8 μg/mL) were also normal for the sex and age of the patient. A test of intravenous arginine hydrochloride showed that the patient did not have GHD (maximum GH peak: 4.6 ng/mL).

Interestingly, the apolipoprotein E (ApoE) genotype of the patient was E4/3, presenting an ApoE4 allele (ε4) that is related to familial hypercholesterolemia, but also with an increased risk of Alzheimer’s disease (AD) or mild cognitive impairment (MCI) [20].

The blood test performed after the second PET-SCAN indicated that there were no significant changes in the previously analyzed parameters, except that the plasma values of IGF-I and IGFBP3 had increased to 185 ng/mL and 3.2 μg/mL, respectively (both within the range of normal values). Therefore, the molar ratio of IGF-I to IGFBP3 increased from 0.04 (initial value) to 0.057 after treatment with GH. Blood glucose remained at normal values (97.3 mg/dL).

The treatment with GH did not produce any side effects, as observed through clinical examinations and blood tests.

## 3. Discussion

In this study, we demonstrated, with PET-SCAN brain images, the positive effect that GH exerts on the human brain in an elderly patient with cognitive deficits and without GHD, particularly in areas related to learning, recent memory, behavior, and visuospatial perception; although, as the images indicate, the metabolic effects of the hormone occurred in practically all cortical areas. In addition, as the patient herself reported, her quality of life and the performance of daily activities improved after treatment with GH, even though the administration of the hormone lasted only three weeks.

The effects of GH on the brain have been previously visualized in a study carried out in patients with GHD, in whom GH was administered for six months [21]. After this time of GH replacement therapy, the neuropsychological tests showed a clear improvement in working memory and cognition, but, perhaps even more importantly, the functional magnetic resonance imaging (fMRI) carried out during the working memory task showed greater activity in several cortical areas and in the right thalamus and the anterior cingulate cortex [21]. A later study, carried out in an older GHD patient with cognitive impairments due to chronic opioid treatment for neuropathic pain, indicated that GH treatment produced clear improvements in visuospatial cognitive functions and a higher metabolism and functioning of the hippocampus, according to what was showed by a proton magnetic resonance spectroscopy study [22]. Our study corroborates these effects, seen with images, of the GH at the brain level, although in our case the patient was without GHD, unlike the aforementioned studies.

Unlike what happens in children and adults with GHD, few studies show that the administration of GH increases cognition in non-GHD human patients with cognitive deficits produced as a result of different pathologies [23,24,25,26,27,28]. However, these positive effects of GH have been widely demonstrated in different experimental animal models [29,30,31,32,33]; even in old animals, in which the cognitive impairment of hippocampal-dependent functions, such as learning and memory, is associated with a decrease in the secretion of GH and IGF-I, as in our species [4,17,18,32].

In the case of the hippocampus, it has been shown to be an important neurogenic niche, where adult neurogenesis takes place in humans [34]. Functionally, the hippocampus is responsible for the acquisition of memory, learning, and recent spatial orientation and navigation (in neurons known as “place cells”). These functions are affected in personality disorders, perhaps due to a continuous excess of glucocorticoids [35], which negatively regulates the formation of new neurons in the subgranular zone (SGZ) of the dentate gyrus of this structure [36].

Recently, it was published that hippocampal neurogenesis begins to decrease abruptly from adolescence to undetectable levels in adults [37]. This led to great controversy, and very recent publications question or contradict these postmortem findings [38,39,40], although there is a possibility that neurogenesis in the adult hippocampus can be deregulated by neurological diseases, such as epilepsy or behavioral disorders [41], which would explain the current divergent opinions about the persistence of adult neurogenesis in man throughout life.

In our study, the metabolic activity of the left hippocampus increased significantly after treatment with GH. This may indicate that treatment with the hormone has induced an increase in the number of neurons in this area, as we and others have shown in rats [10,11]. Another possibility is that GH has induced the outbreak of dendritic spines and changes in the length and density of pre-existing dendrites in the hippocampus, as has been shown to occur after intracerebroventricular administration of the hormone in adult rats [42]. If we could have used 3′-deoxy-3′-[18F] fluoro-l-thymidine instead of FDG, we could have detected whether the changes observed in PET-SCAN were due or not to adult neurogenesis, as a study has shown in adult rats [43].

Interestingly, hippocampal atrophy (measured by MRI) is an early marker of AD that correlates with memory disturbances [44], and it has been found that cerebral glucose metabolism is significantly reduced in early stages of this disease [45]. In addition, the ApoE genotype of the patient was ε4/3, and it has been described that the presence of a single copy of the ε4 allele increases the risk of developing AD, MCI, or other cerebral pathologies with cognitive impairment [20], although the main risk of development of AD occurs in homozygous individuals showing two ε4 copies (ε4/4) [20]. The patient fulfilled two of the core clinical criteria for the diagnosis of MCI, such as the concern for a change in cognition and deterioration in one or more cognitive domains [46]. Although we did not analyze whether there was an accumulation of β-amyloid that accompanied the cognitive deterioration existing in the patient, as typical characteristics of AD, it seems unlikely that we were facing an early stage of this disease, because the decrease in metabolism in the left hippocampus could only be detected after the quantitative analysis performed with Neurocloud and there was no decrease in the temporoparietal acquisition of FDG. The patient will be studied again in the next months to analyze if the positive results obtained with the GH treatment continue. In any case, it has recently been postulated that a treatment with GH could be useful in this neurodegenerative disease [47,48].

We cannot discard the observation that GH has induced neurogenesis in the hippocampus of the patient described in this study, thus improving her recent memory; but it is very unlikely, or practically impossible, that adult neurogenesis has been the cause of the increase in glucose metabolism in virtually all cerebral cortical areas, due to the low number of neurogenic niches described in the adult human brain and their progressively lower activity as we get older.

Other regions of interest in which hypometabolism was observed before treatment with GH were the left amygdala, the left parahippocampus, the left cuneus, and the subgenual area of the anterior cingulate cortex.

The amygdala is involved in emotional responses (pleasure, fear, anger, anxiety), but it also determines how emotions adhere to memories, mainly forming new memories related to fear, although a recent article describes fear as the result of a very complex memory network [49]. A more recent study reports that in the basolateral amygdala of adult mice, there are neurogenic precursor cells that give rise to newly formed interneurons [50]. However, adult neurogenesis in the human amygdala has not yet been demonstrated; therefore, it is unlikely that the increased metabolism observed in this structure after the treatment with GH could be due to adult neurogenesis.

As for the parahippocampus, recently it has been described that, in humans, its posterior section is involved in visuospatial perception, while the previous section is related to mnemonic processes, suggesting that this structure acts as a functional interface between perception and memories [51]. The treatment with GH also improved the low metabolic activity previously observed in this structure, but the changes, in this case, did not reach statistical significance.

The cuneus receives information from the upper area of the contralateral retina, which represents the lower visual field. Its function is basic visual processing, related to attention and working memory. In this case, the existence of left hypometabolism before treatment with GH was detected after the Neurocloud PET analysis, showing a clear asymmetry between the left and the right side of this structure. Treatment with GH also led to an increase in the acquisition of the left side that was now within the range of values for the normal population of the database used, but also on the right side; therefore, the asymmetry between both sides continued to exist, as shown in Table 2.

In the case of the subgenual area of the anterior cingulate cortex, there was a clear asymmetry between the left and right side before administration of GH, but this disappeared after treatment with the hormone, with the metabolism already normal on both sides. This area has been related to depression [52] and its affectation is related to mood disorders [53], which may explain some of the symptoms observed in our patient. On the other hand, structural and functional anomalies in this region have been associated with major depressive disorders, mainly when they are accompanied by a decreased volume of both temporal lobes and the left hippocampus and parahippocampus [54], but this was not the case in the patient described here.

The brain uses mainly glucose to obtain the energy needed to function correctly [55]. A recent study describes that there is a significant correlation between the cerebral metabolic rate of glucose, measured by FDG PET, and the level of consciousness in patients in a vegetative or minimally conscious state, with the metabolic rate of glucose being capable of differentiating between both conditions [56].

An elegant study carried out in a model of dwarf rats specifically deficient in GH and IGF-I showed that in these animals, there was a marked decrease in glucose metabolism in many areas of the brain, particularly those involved in learning and memory, dependent on the hippocampus, indicating that the decrease in GH/IGF-I production associated with aging plays an important role in the evolution towards an elderly brain [57]. Moreover, in that study, it was seen that the production of ATP in the hippocampus was decreased by 15%, which contributed to the statement that GH and IGF-I play a clear role in the regulation of glucose use and cerebral energy metabolism [57]. The same happens in man: aging leads to a series of brain deficits, such as learning and memory, neurogenesis, synaptic density, and modifications in dendrite architecture (see [57] for review). In addition, it is logical that a decrease in the production of GH/IGF-I alters the rate of metabolic renewal of important neurotransmitters, such as acetylcholine and noradrenaline, given the important role they play in the hypothalamic regulation of the synthesis and release of GH [58].

These age-related cerebral deficits have been shown to be reversed by chronic infusion of IGF-I into the lateral ventricle of aged rats [59], or by GH treatment in very old rodents [60], and more recent studies in man indicate that high plasma levels of GH and IGF-I maintain the functional quality of the working memory during aging [61]. However, the patient we treated was without GHD, and the plasma levels of IGF-I were within normal values before treatment with GH. Therefore, her MCI and the hypometabolisms found in the first PET-SCAN cannot be explained by a normal process of aging associated with a deficient functioning of GH/IGF-I.

Cerebral glucose hypometabolism has been linked to the early stages of AD [62]; in addition, the genotype of the patient presents a copy of the ε4 allele, which implies an increased risk of developing AD [20], but also a decrease in the cerebral metabolism of glucose [63]. A recent study in mice, genetically modified to carry one of the three human ApoE alleles in place of their normal ApoE gene, demonstrated that the ApoE3 and ApoE4 brains of these animals showed a significant reduction in the expression of molecules involved in IGF-I signaling, namely IGF-I itself, Irs 1 (insulin receptor substrates), and the Glut4 glucose transporter [64]; the result is a reduction in glucose uptake by the brain. In the same study, it was shown that ApoE4 brains had lower levels of Pparg, a nuclear receptor that regulates neuronal survival [65] and mitochondrial biogenesis [66]. These findings may explain the reduction of glucose uptake and deficient energy production in the brain of subjects with a ε4 allele, as well as may explain the cognitive and metabolic deficits observed in our patient and the recoveries (cerebral metabolic activity and cognitive functions) observed after treatment with GH. As indicated above, GH induces the local expression of IGF-I in the human fetal cortex [19], and there is the possibility that this effect also takes place in the adult brain, but has not yet been seen. GH increases the production of IGF-I in the liver and, consequently, the plasma levels of this peptide, as it happened in this study. However, unlike what happens with GH, only a small fraction (approximately 30%) of circulating IGF-I [free IGF-I], crosses the blood–brain barrier. Therefore, instead of considering the total IGF-I, we must analyze the IGF-I/IGFBP3 molar ratio, which in this study, only increased from 0.04 to 0.057 after treatment with GH, an amount that does not seem to be high enough as to attribute the changes observed in the brain to the circulating IGF-I. Hence, if the effects we observed in this study were produced by IGF-I, this must have occurred due to the induction exerted by GH on the synthesis of that peptide in the brain. IGF-I signaling implies the activation of PI3K/Akt pathways, and phosphorylated Akt induces the translocation of Glut4 vesicles to the plasma membrane for allowing the entry of glucose into the cells [67]; but the activation of PI3K/Akt is also a key signaling pathway for GH actions, as our group demonstrated [68,69]. Therefore, a direct effect of GH on brain metabolism seems to be the most feasible explanation for the results obtained in this study. In addition, GH may have produced an increase in blood flow to the brain, which would allow increased uptake of FDG. Recently, we described that GH induces a significant reparative effect on the endothelial dysfunction that appears after atherogenic stimuli, such as hypercholesterolemia [70]; moreover, GH is a mitochondrial protector [26,71], and atherogenesis is related to oxidative stress [70]. Given that the patient we treated had high levels of cholesterol and triglycerides in plasma, it is also possible that, despite the short time of treatment, GH may have contributed to improving blood supply to the brain, facilitating the uptake and metabolism of glucose and producing the changes described here (both in terms of PET-SCAN images and cognitive tests).

GHRH (growth hormone-releasing hormone) is a major inducer of the synthesis and secretion of GH in the pituitary gland [58] and perhaps in other territories [4]. A randomized, double-blind, placebo-controlled trial analyzed the effects of a synthetic analogue of GHRH (Tesamorelin; Theratechnologies Inc., Montreal, QC, Canada) administered subcutaneously (1 mg/day) for 20 weeks in 61 adults with MCI and 76 healthy adults. The results obtained showed that this GHRH analogue had positive effects on cognition in both groups studied [72]. An additional clinical trial of the same group carried out on 30 adults (age 55–87 years, 17 with MCI), using the same GHRH analog at the same doses and time, showed that this treatment significantly increased gamma-aminobutyric levels acid (GABA) in three brain regions of the left side (frontal dorsolateral, posterior cingulate, and posterior parietal), increased *N*-acetylaspartylglutamate in the frontal cortex, and decreased myoinositol (an osmolyte related to AD) in the posterior cingulate, inducing a positive effect on cognition in both groups of participants without affecting the regulation of plasma glucose [73]. However, in that study, no changes were found in brain glutamate levels, unlike what has been reported in preparations of hippocampal cuts from old rats treated with GH or IGF-I [32], but consistent with the effects of GH on the density and functionality of GABAB receptors in male rats in areas of the brain related to cognition [74]. These data seem to support our findings using GH in this study because although it cannot be discarded that GHRH exerts its own effects at the brain level, as it seems to do in other territories, it is clear that the administration of GHRH or one of its analogues induces the release of GH [75].

In summary, for the first time, we demonstrated with FDG PET-SCAN the positive effects that GH exerts on the metabolic activity of the brain of a non-GHD older woman with MCI and dyslipidemia. Our data do not allow us to verify if the existing MCI was due to the presence of the ε4 allele or dyslipidemia, or both, but it is clear that the GH treatment significantly improved the patient’s cognitive disabilities. This improvement still continues, three months after the treatment has been interrupted, and corroborates our previous data in patients and animal models with cognitive deficits produced by acquired brain damage after being treated with GH.

## 4. Materials and Methods

The patient was a 61-year-old Caucasian woman, whose father had died of a very aggressive AD at 64 years of age, and who had also suffered from type II diabetes very poorly controlled for years. Her family history also included that an older sister had begun to present important cognitive alterations, at the age of 59, and frequent episodes of absences for unknown reasons that required antiepileptic treatment.

The patient had had four children, all healthy; she had a degree in marketing and was working as a director of a company for 35 years. She had no toxic habit, although she had smoked 20 cigarettes a day from 18 to 35 years of age. The only clinical information of interest is a hysterectomy because of a fibromyoma, and familial dyslipidemia for which she never wanted to be treated with medications because she claimed that her diet was very healthy and rich in omega-3 fatty acids. Her only medication was melatonin (50 mg/day, orally) that she had been taking for eight years before going to sleep.

Upon admission to our medical center, the patient reported that in the last two years, she had suffered significant stress due to work problems, a decrease in recent memory, sporadic episodes of disorientation in time and space, and some behavioral alterations. These affectations were confirmed by her husband. Due to the illness that her father had suffered, she was afraid that AD would also begin to develop in her, even though she was totally independent in the activities of daily life, and had an intense work activity and quite a rich social life.

The clinical examinations were normal. The patient expressed herself with fluency and richness of language. The blood pressure was 135/70 mm Hg. In spite of her dyslipidemia, her cardiologist never detected cardiac or vascular affectations. The body mass index (BMI) was normal: 23 kg/m^2^. Before starting treatment with GH and one week after finishing it, a blood test was performed (hematimetry, biochemistry, thyroid hormones, cortisol, IGF-I, IGFPB3, and tumor markers). To evaluate the possibility of a GHD, a typical arginine test (30 g of intravenous infusion of arginine hydrochloride between 0 and 30 min) was performed during the first blood test, and samples were taken to analyze plasma levels of GH at times 0, 30, 60, 90, and 120 min.

In a blood sample, the ApoE genotype of the patient was analyzed by molecular hybridization with amplification PCR (polymerase chain reaction).

The studies and treatment were carried out according to the protocols of the Foltra Medical Center in accordance with Spanish legislation for the use of GH off-label and the Code of Ethics of the World Medical Association (Declaration of Helsinki).

Before the GH treatment, the signed informed consent of the patient was obtained to be treated with the hormone and to allow the results obtained could be published. The study was approved by the Ethical Committee of the Foltra Medical Center (Fol2018-002).

**Figure 4:**
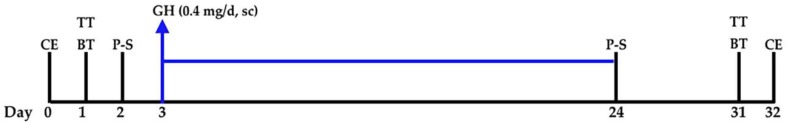
After the clinical examination (CE, day 0), a blood test (BT: hematimetry, biochemistry, hormones, and plasma tumoral markers) was performed; day 1. In the same day, a Tavec test (TT) was carried out. One day later, a FDG-PET-SCAN (P-S) was carried out; day 2. The next day (day 3), the patient began to be treated with GH for 21 days (0.4 mg/day, subcutaneously (sc), blue arrow and blue line). Twenty-one days later, a new FDG-PET-SCAN was performed just 1 h after the last administration of GH (day 24). Seven days later, a new BT and a TT were performed (day 31), and one day after this, a new clinical examination was performed.

Figure 4 shows the sequence of examinations and treatment carried out.

### 4.1. Cognitive Tests

Initially, a TAVEC verbal learning test was performed. This test is based on other similar psychometric tests, such as the test of 15 words of Rey or the more recent California verbal learning test (CVLT). In all of them, the verbal element learning test is used. In the TAVEC test specifically, three lists are used for Learning, Interference, and Recognition. The test allows to establish the normality of the patient (in comparison with a similar sample in age, sex, and educational level), and describe the functioning of the patient’s memory and determine the form and reasons for its deviation (if any).

One month after the first test, a new TAVEC test was performed.

### 4.2. PET-SCANs

After the first TAVEC test, the metabolic activity of the patient’s brain was analyzed by PET-SCAN images in cross sections acquired 30 min after the administration of fluorodeoxyglucose (FDG) 4.2 mCi 18-F. One day later, and once the signed informed consent was obtained, the patient began treatment with GH (0.4 mg/day, subcutaneously, at 10.00 h; Nutropín, Ipsen, Spain).

Twenty-one days later, a new PET-SCAN (requested by the patient) was performed under the same conditions, with the exception that the dose of FDG administered was slightly lower (3.6 mCi) than the previous one.

The last GH administration took place 1 h before the second PET-SCAN was performed. Both PET-SCANs were performed at 11.00 h.

In both cases, the patient was fasting from 14 h before the PET-SCAN.

### 4.3. Statistical Analysis

Due to the short time elapsed between the two TAVEC tests, no statistical analysis of the results obtained between the two tests was performed.

Both PET-SCANs were quantified by using Neurocloud PET software (Qubiotech Health Intelligence, A Coruña, Spain), which provides both ROI (regions of interest)-based statistical analysis using the regions on the Hammersmith Atlas [76] and voxel-based “statistical parametric mapping” (SPM), both comparing the patient images with a database of 97 healthy subjects. This allows evaluation of the values obtained in any subject in terms of percentage of standard deviations (SD) with respect to the normal population, and from this, the statistical significance of the data may be obtained. This software provides embedded spatial and intensity normalization methods. Spatial normalization is performed by a 12-parameter affine registration between the patient PET and a template on the MNI (Montreal Neurological Institute) space. Intensity normalization is performed in a ratios histogram fashion.

The software provides a proprietary database with 97 FDG-PET acquisitions performed on pretreatment oncologic patients, in which an acquisition was added using the standard brain protocol before whole body acquisition. All patients underwent a neurological examination and signed informed consent to be included in the trial. All the images in the database were visually verified and reported as normal by three independent nuclear medicine specialists. The studies carried out in these patients consisted of MRI T1 3D and -FDG-PET (brain protocol). As criteria for normality, the following were established: (1) no detection of cerebral atrophy in MRI; (2) three independent reports from three specialists in nuclear medicine, which affirmed the nonexistence of brain anomalies in the FDG-PET; (3) an absence of neurological or psychiatric history and normal neurological examination (MMSE (Mini-mental state examination) normal scores: 25–30).

The statistical significance of the data was established for a value of *p* < 0.05.

## 5. Conclusions

The results of this study clearly show, for the first time with cerebral images in an older woman with MCI and without GHD, that GH exerts a strong and positive effect on cortical cerebral structures. Of special interest is the fact that the hormone increases metabolic activity in areas related to memory and cognition. Although this is only a case report and more studies are needed and with more patients, there is a possibility that a treatment with GH in MCI or in the early stages of Alzheimer’s disease may be useful to delay the progression of this disease and/or other neurodegenerative disorders that lead to cognitive alterations.

## Figures and Tables

**Table 1:** Results obtained in the TAVEC test performed in basal conditions (Pre) and 1 month later (seven days after finishing the treatment with GH). The values in the first and second tests correspond to Z-scores (the average value for a normal population is 0). The scores in the first test were, in general, lower (highlighted in blue color) than the average of the normal subjects, indicating a mild cognitive deficit. However, the second test indicates that most of the results obtained are now in the average range for normal subjects or even higher than the average.

ASSAY	ASSAY	Pre	1 Month
1. RI-A1	Immediate recall of the first learning assay	−1	0
2. RI-A5	Immediate recall of the fifth learning assay	−2	0
3. RI-AT	Total words remembered in the whole of the 5 assays	−2	0
4. RI-B	Immediate recall of the list of interference	−1	1
5. RG-Pr	Percentage of words in the region of primacy, over the total number of words remembered in the total of the 5 tests	−2	0
6. Rg-Rc	Percentage of words from the middle region, about the total number of words remembered in the 5 essays	0	0
7. Rg-Rc	Percentage of words from the region of recency, on the total number of words remembered in the 5 assays	−1	0
8. RL-CP	Short-term free memory	−1	1
9. RC-CP	Long-term free memory	−1	0
10. RL-LP	Memory with short-term keys	−1	0
11. RC-LP	Memory with long-term keys	−2	0
12. Esem-RI-A	Use of the serial strategy in the immediate recall of list A	−1	−1
13. Esem-RI-S	Use of the serial strategy in the immediate recall of list B	−1	−1
14. Esem-RL-CP	Use of serial strategy in short-term free recall	−1	0
15. Esem-RL-LP	Use of serial strategy in long-term free recall	−1	−1
16. Eser-RI-A	Use of the semantic strategy in the immediate recall of list A	−1	−1
17. Eser-RI-B	Use of the semantic strategy in the immediate recall of list B	−1	0
18. Eser-RL-CP	Use of the semantic strategy in short-term free recall	0	0
19. Eser-RL-LP	Use of the semantic strategy in long-term free recall	1	0
20. P	Total number of perseverations	1	1
21. I-RL	Number of intrusions in the whole of free recall tests	0	1
22. I-RL	Number of intrusions in the whole of memory tests with keys	−1	0
23. Recon-Ac	Number of success in the recognition test	−2	1
24. FP	Number of false positives in the recognition test	1	0
25. Discriminability	Discrimination index	−2	0
26. Bias	Response bias index	0	0
27. RI-S versus R1-A1	Comparison between the memory of list B and the memory of the first learning test in list A	0	1
28. RL-CP versus RI-A5	Comparison between short-term free recall and the immediate recall of the fifth learning test in list A	−1	0
29. RC-CP versus RO-LP	Comparison between remembering with short-term keys and remembering with long-term keys	1	0
30. RL-LP versus RL-CP	Comparison between long-term free memory and short-term free memory	1	0
31. RC-LP versus RL-LP	Comparison between memory with long-term keys and long-term free recall	0	1
32. Recon-Ac versus RL-Lp	Comparison between recognition and long-term free recall	−1	0
33. Recon-Ac versus Rcl-LP	Comparison between recognition and recall with long-term keys	−1	0

**Table 2:** Quantitative analysis of the metabolism (Neurocloud PET) in the regions of interest expressed as a percentage of deviation with respect to the normal population of the database, in the first and second PET-SCAN performed. The values highlighted in blue indicate a standard deviation <1.5 with respect to the mean of the normal population. L: Left side; R: Right side; Asym: Asymmetry; Hypom: Hypometabolism; Bilat: Bilateral; AC/SGA: Subgenual area of the anterior cingulate cortex. *p*: Statistical significance of each hypometabolism with respect to the normal population.

ROI	First PET-SCAN				*p* <	Second PET-SCAN			
	L	R	Asym	Hypom		L	R	Asym	Hypom
Hippocampus	−12.49	−6.55	−5.94	Left	0.025	−6.49	−2.84	−3.65	
Amygdala	−13.38	−7.20	−6.18	Left	0.025	−9.47	−1.52	−7.96	
Parahippocampus	−11.93	−7.24	−4.69	Left	0.025	−10.86	−8.19	−2.67	Bilat
Cuneus	−5.42	4.06	−9.48	Left	0.025	−0.90	6.41	−7.31	Left
AC/SGA	−2.43	−11.16	8.73	Right	0.025	3.08	0.91	−2.17	
